# Percutaneous Image-Guided Cryoablation of Endophytic Renal Cell Carcinoma

**DOI:** 10.1007/s00270-023-03633-5

**Published:** 2024-03-14

**Authors:** Christian Greve Jensen, Marco Dybdahl, John Valtersson, Bo Redder Mussmann, Louise Aarup Duus, Theresa Junker, Pia Iben Pietersen, Lars Lund, Brian T. Welch, Ole Graumann

**Affiliations:** 1 Faculty of Health Sciences, Medicine, University of Southern Denmark (SDU), Odense, Denmark; 2 Research and Innovation Unit of Radiology - UNIFY, SDU, Odense, Denmark; 3 Department of Radiology, OUH, Odense, Denmark; 4 Faculty of Health Sciences, Oslo Metropolitan University, Oslo, Norway; 5 Department of Urology, OUH, Odense, Denmark; 6 Department of Radiology, Mayo Clinic, Rochester, MN USA; 7 Department of Radiology, Aarhus University, Arhus, Denmark; 8 Aarhus University, Arhus, Denmark

**Keywords:** Renal cancer, RCC, Endophytic tumor, Cryoablation, PCA, Percutaneous, Ablation

## Abstract

**Purpose:**

Endophytic renal cancer treatment is a challenge. Due to difficulties in endophytic tumor visualization during surgical extirpation, image-guided percutaneous cryoablation (PCA) is an attractive alternative. The minimally invasive nature of PCA makes it favorable for comorbid patients as well as patients in which surgery is contraindicated. Oncological outcomes and complications after PCA of endophytic biopsy-proven renal cell carcinoma (RCC) were reviewed in this study.

**Materials and Methods:**

Patients were included after a multidisciplinary team conference from January 2015 to November 2021. Inclusion criteria were endophytic biopsy-proven T1 RCC treated with PCA with one year of follow-up. Complications were reported according to the Cardiovascular and Interventional Radiological Society of Europe (CIRSE) classification system and the Clavien-Dindo classification (CDC) system. Major complications were defined as a grade ≥ 3 according to the CDC.

**Results:**

Fifty-six patients were included with a total of 56 endophytic tumors treated during 61 PCA sessions. The median RENAL nephrometry score was 9 (IQR 2), and the mean tumor size was 25.7 mm (SD ± 8.9 mm). Mean hospitalization time was 0.39 (SD ± 1.1) days. At a mean follow-up of 996 days (SD ± 559), 86% of tumors were recurrence free after one PCA. No patients progressed to metastatic disease. According to the CIRSE classification, 10.7% (*n* = 6) had grade 3 complications, and 5.4% (*n* = 3) had CDC major complications.

**Conclusion:**

This study demonstrates that PCA of endophytic biopsy-proven T1 RCC is safe with few major complications and excellent local tumor control rates at almost three-year mean follow-up.

**Level of Evidence 3:**

Retrospective cohort study.

**Graphic Abstract:**

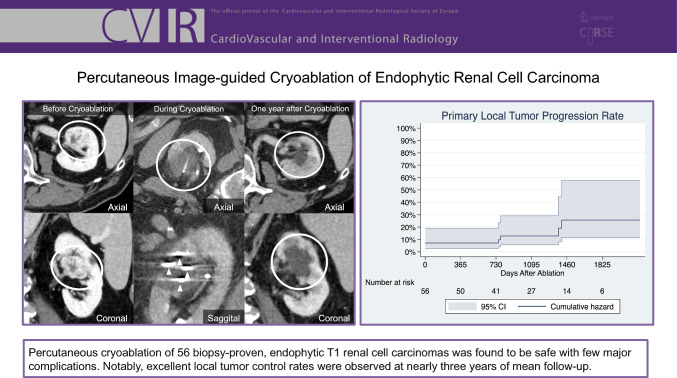

**Supplementary Information:**

The online version contains supplementary material available at 10.1007/s00270-023-03633-5.

## Introduction

In the last decade, global renal cell carcinoma (RCC) rates have surged, now representing 2.2% of worldwide cancer cases, resulting in 70,000 annual deaths in Europe and North America [1].

Due to the increasing use of cross-sectional imaging, the incidence of RCC is expected to rise, thus resulting in an increased number of RCC treatments. Therefore, efficient and effective treatments are warranted for all types of RCC [2].

The 2022 European Association of Urology (EAU) guidelines conditionally recommend percutaneous cryoablation (PCA) for patients with T1a RCC tumors (< 4 cm), particularly for elderly or comorbid individuals [3]. PCA can be successfully applied in older, comorbid patients with impaired renal function. PCA can be performed in patients with bilateral tumors, a solitary kidney, and those who are deemed unsuitable for surgery under general anesthesia [3–6]. Endophytic RCC tumors present a challenge, often requiring radical nephrectomy due to difficulties in surgical excision, tumor identification, and margin assessment [7]. However, PCA is emerging as a potential solution for patients with endophytic lesions, as recent studies have reported encouraging oncological outcomes and low complication rates [8–10]. Nonetheless, more comprehensive, long-term investigations are needed to assess the effectiveness and safety of PCA in managing endophytic T1 RCC. This retrospective cohort study aims to describe oncological efficacy and safety in a cohort of patients receiving PCA for endophytic RCC at anonymized hospital from January 1, 2015, to November 1, 2021.

## Materials and Method

This study was conducted in accordance with the Strengthening the Reporting of Observational Studies in Epidemiology (STROBE) statement [11].

### Setting

**Fig. 1:**
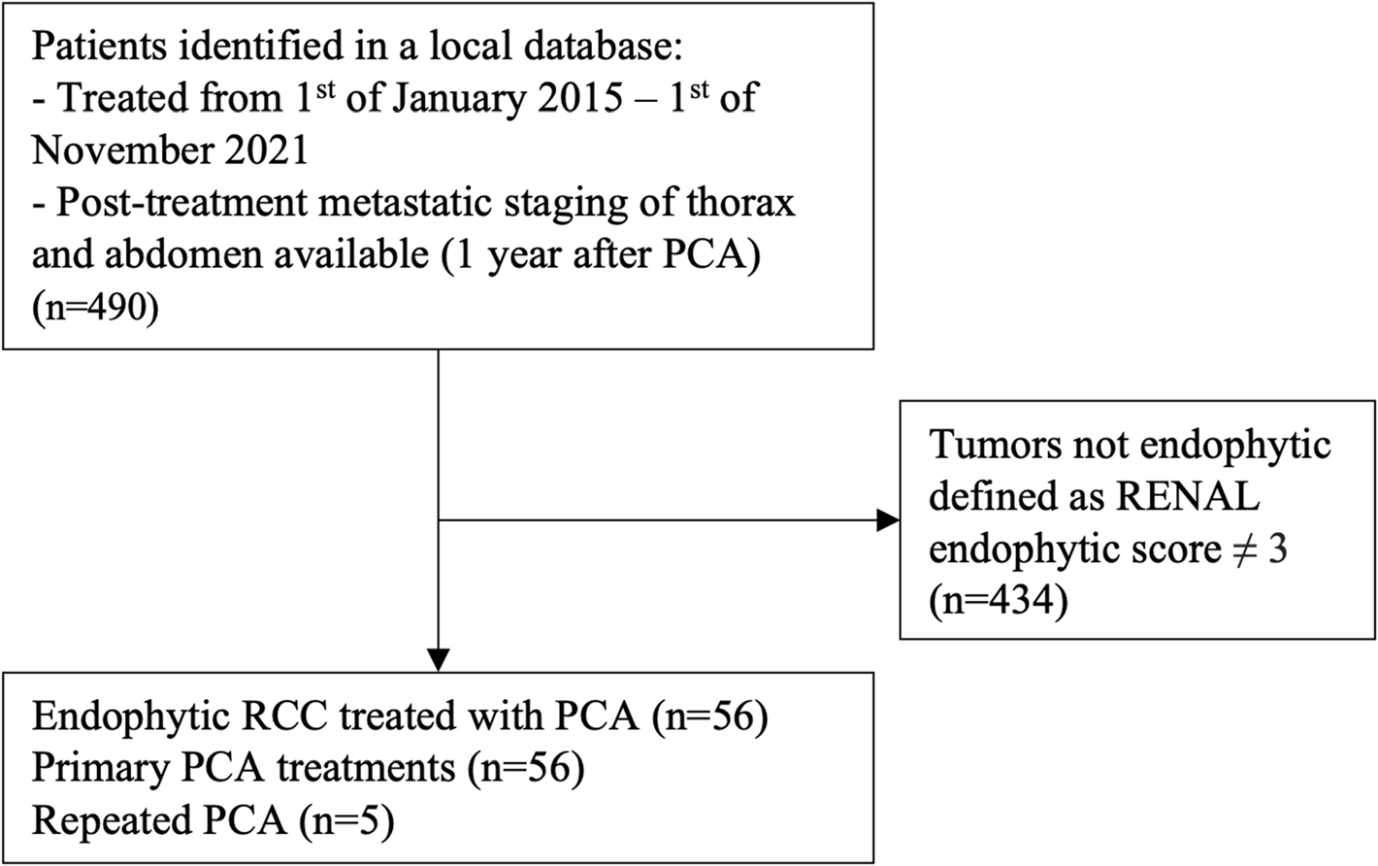
Flowchart showing patient selection. ^a^Percutaneous cryoablation (PCA). ^b^Radius–endophytic–nearness–anterior–location (RENAL). ^c^Renal cell carcinoma (RCC)

Patients were retrospectively included from anonymized hospital (Fig. 1). Treatment with PCA was discussed at a multidisciplinary team conference. Before PCA, all tumors were biopsy-verified as RCC. Diagnostic imaging of the abdomen and chest was carried out to ensure the absence of metastases. All PCA procedures were performed by two senior interventional radiologists with three and seven years of experience with PCA, respectively. Following PCA, the patient was observed for up to four hours before discharge from the hospital. If needed, patients were hospitalized. Full description of PCA is available as Online Resource 1.

Tumor complexity was determined before PCA using radius–endophytic–nearness–anterior–location (RENAL) score evaluated by a senior radiologist (xx) with 17 years of experience [12]. Prior to PCA, all patients had an American Society of Anesthesiologists (ASA) score determined by the anesthesiologist assessing the patient prior to PCA. eGFR was calculated using the CKD-EPI equation [13].

### Follow-Up

Follow-up data were collected until the December 1, 2022. Standard follow-up diagnostics included multiphase contrast-enhanced CT (CECT) of kidneys performed after 3 and 6 months, followed by repeated yearly CECT scans of thorax and abdomen with multiphase contrast of the kidneys for 5 years. Patients with impaired renal function had a non-contrast abdominal MRI performed, supplemented with contrast-enhanced ultrasonography (CEUS) of the ablation cavity and a non-contrast CT of the chest. If residual tumor was suspected, either a biopsy of the enhancing lesion was performed for verification or active surveillance with diagnostic imaging was performed in 3- to 6-month intervals.

Follow-up was terminated if the patient:

- Finished the planned 5-year follow-up
- Died
- Was treated for recurrence or incomplete ablation by other means than PCA
- Decided to withdraw from treatment
- Moved away from the hospital catchment area

### Oncological Outcomes

Technique efficacy was reported using the terminology suggested by Ahmed et al. [14].

*Incomplete ablation/residual unablated tumor* was defined as an enhancement in the ablation cavity detected at the first follow-up after the procedure or if residual tumor was suspected at the first follow-up scan and confirmed at the next scan or by biopsy.

*Local tumor recurrence* was defined as nodular enhancement in the ablation cavity found in follow-up after the initial scan showed no residual tumor.

Treatment outcomes after one procedure were described as the *primary efficacy rate* and *primary local tumor progression rate*. The *secondary efficacy rate* and *secondary local tumor progression rate* were defined as outcomes after one or more treatments.

### Complications

Complications in this study were defined according to the Cardiovascular and Interventional Radiological Society of Europe (CIRSE) classification for interventional radiology complications [15] as well as the Clavien-Dindo classification (CDC) of surgical complications [16]. Both complication grades were included to ensure comparability with previous studies. Major complications were defined as a grade ≥ 3 according to the CDC. Periprocedural complications, complications during hospitalization post-treatment, and complications that appeared during follow-up were recorded.

Hospitalization time after PCA or readmission due to complications was defined as nights of hospitalization.

### Statistical Analysis

Continuous variables were described as mean with ± standard deviation (SD) or median with interquartile range (IQR), depending on the empirical distributions tested with Shapiro–Wilk test for normality.

Categorical variables were described with percentages and frequencies.

Furthermore, an Aalen–Johansen cumulative hazard estimate was performed. Patients were censored at the last follow-up scan available. Incomplete ablation and local tumor recurrence were defined as treatment failure.

All analyses were conducted using “STATA 17BE” (StataCorp, College Station, TX, USA).

## Results

No patients had prior history of ipsilateral kidney surgery. One patient had a horseshoe kidney. Additional baseline characteristics are presented in Table 1.

**Table 1:** Baseline characteristics

*Patients*	
Gender (*n* = female/male)	15/41
Age at intervention, mean (SD)	61.5 (± 11.9)
ASA^a^ score, mean (SD)	2.1 (± 0.7)
Pre-procedure eGFR^b^, mL/min mean (SD)	81.8 (± 18.5)
*Tumors*	
Tumors, *n*	56
Tumor size (mm), mean (SD)	25.7 (± 8.9)
RENAL^c^ score, median (IQR)	9 (2)
*Tumor histology*	
Clear cell, *n* (%)	41 (73)
Papillary, *n* (%)	8 (14)
Chromofobe, *n* (%)	5 (9)
Unclassified RCC, *n* (%)	2 (4)
*Procedures*	
Procedures, *n*	61
Anesthesia (general anesthesia/sedation)	16/45
Operative time in minutes, median (IQR)	63 (21)
Hydrodissection *n* (%)	22 (39)
No. of cryoprobes median (IQR)	3 (1)

^a^American Association of Anesthesiology

^b^Estimated glomerular filtration rate (eGFR), by the CKD-EPI equation

^c^Radius–endophytic–nearness–anterior–location (RENAL)

^d^Renal cell carcinoma (RCC)

In total, 7% (*n* = 4) of the treatments were incomplete. Local recurrences were seen at 754, 777, 1373, and 1406 days, respectively, corresponding to 7% (*n* = 4). One patient was not fit for further diagnostics and treatment due to extensive comorbidities. Two patients were treated with partial nephrectomy (PN) after detection of unsuccessful primary PCA.

Five patients underwent repeat PCA, and four of these were successfully treated, resulting in a primary and secondary efficacy rate of 86% and 93% at a mean follow-up of 996 (SD ± 559) and 1022 days (SD ± 531), respectively (Table 2).

**Table 2:** Oncological and safety outcomes

Days of imaging follow-up *not* including follow-up time after repeat PCA^a^ mean (SD)	996 (± 559)
Days of imaging follow-up including follow-up time after repeat PCA mean (SD)	1022 (± 531)
Primary efficacy rate % (*n*)	86% (48)
Secondary efficacy rate % (*n*)	93% (52)
Incomplete ablations % (*n*)	7% (4)
Local recurrence % (*n*)	7% (4)
Nights of Hospitalization, mean (SD)	0.39 (± 1.1)
Survival at latest follow-up % (n)	91% (51)
eGFR^b^ change, mean (SD)	− 2.2 (± 7.9)^c^

^a^Percutaneous cryoablation (PCA)

^b^Estimated Glomerular Filtration Rate (eGFR), by the CKD-EPI equation

^c^Two patients had no postoperative creatinine measurement available and was not included in this measurement. Mean time between measurements is 114 days

**Fig. 2:**
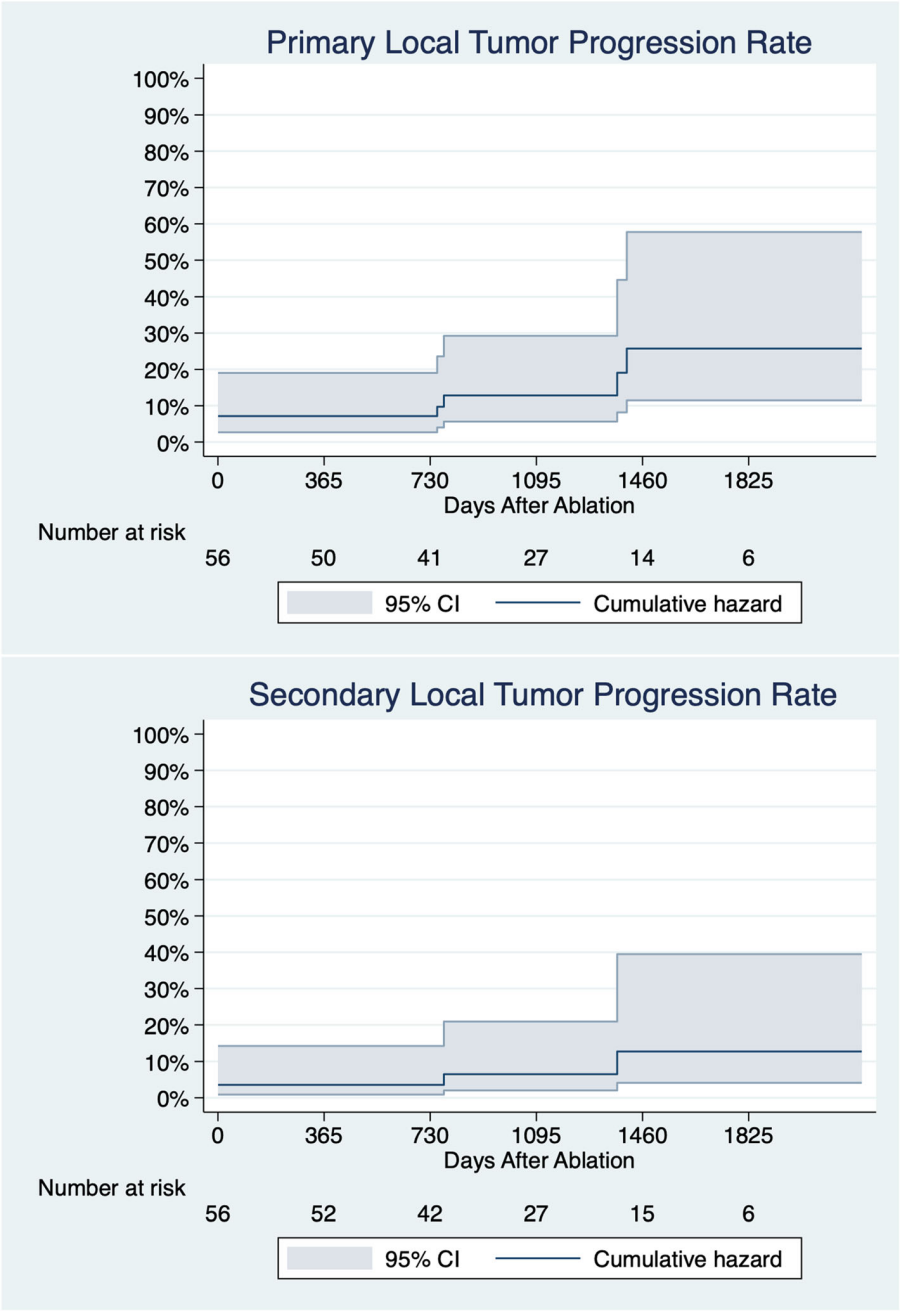
Cumulative incidence estimates of local tumor progression

The Nelson–Aalen cumulative incidence estimates of primary local tumor progression rate were 7.1%, 12.8%, and 25.8% at 1, 3, and 5 years, respectively. The secondary local tumor progression rates were 3.6%, 6.5%, and 12.8% at 1, 3, and 5 years, respectively (Fig. 2).

No metastasis related to RCC was observed in any of the 56 patients included.

Five patients died during the follow-up period: two from disseminated prostate cancer, one from disseminated colon and gastric cancer, one from ischemic heart disease, and lastly, one patient died from gastrointestinal bleeding caused by alcoholic liver cirrhosis. No deaths were related to RCC or PCA.

Major complications were seen in 5.4% (*n* = 3). Two patients with iatrogenic pneumothorax were treated along with the PCA procedure with a 7F chest drain. Both drains were removed before the patients were woken from general anesthesia, and both patients left the hospital the same day without further complications. One patient went into cardiac arrest after extubation and was admitted to the intensive care unit (ICU) for monitoring. This patient was discharged two days later, with no long-term sequelae.

CIRSE grade 3 complications were experienced by 10.7% (*n* = 6). Complication details are delineated in Table 3.

**Table 3:** Safety, complications, and hospitalization

Complication	*n* (%)	CIRSE^a^	CDC^b^	Description
Iatrogenic pneumothorax (*n*)	2 (4)	1	3b	Both treated within procedure
Hematoma (*n*)	2 (4)	3	2	3 and 5 nights of hospitalization
Subcapsular bleeding (*n*)	1 (2)	3	1	5 nights of hospitalization
Cardiac arrest (*n*)	1 (2)	3	4	Cardiac arrest after extubating, admitted to ICU^c^ for 3 days, discharged in habitual state
Pain and hematuria (*n*)	2 (4)	2	1	Both readmitted, 1 night of hospitalization
Small abscess in cryocavity (*n*)	1 (2)	3	2	Treated with antibiotics
Urinary retention (*n*)	1 (2)	3	1	Reason unknown, 3 nights of hospitalization, conservatively treated
Infection of insertion site	1 (2)	0	2	Treated with antibiotics
Post Ablation Syndrome	10 (18)	0	1	Post Ablation Syndrome, no treatment
Pain	2 (4)	0	1	No reason found

^a^Cardiovascular and Interventional Radiological Society of Europe (CIRSE)

^b^Clavien Dindo Classification of Complications (CDC)

^c^Intensive care unit (ICU)

## Discussion

This single-center study investigates the oncological and safety outcomes in a larger cohort of patients with biopsy-proven endophytic RCC treated with PCA. We demonstrate the use of PCA to treat endophytic RCC is associated with a promising primary efficacy rate of 86%. Notably, none of the patients in this cohort developed metastatic RCC during the almost three-year mean follow-up period. Furthermore, overall rate of major complications was low.

Only a few studies investigating oncological outcomes after PCA of endophytic RCC have been published. This study found Nelson–Aalen cumulative incidence estimates of primary local tumor recurrence, which were 12.8% at 3 years and 25.8% at 5 years, respectively. These findings are consistent with the results reported by Murray et al. [8], who observed comparable outcomes of 10% at 3 years and 25% at 5 years in their RCC sub-cohort (*n* = 25) . The cohort and technical execution of PCA in our study closely resemble those in the study by Murray et al. However, the tumors selected for our study were defined as endophytic according to the RENAL classification, while the study by Murray et al. defined endophytic tumors as those covered by the renal cortex, making them more central than those included in our study.

In a study by Autrusseau et al. [9], 14 patients with centrally placed RCC were treated with PCA, along with concomitant balloon occlusion of the renal artery. Among the patients, 14% (*n* = 2) experienced local tumor progression; one treatment was incomplete, and another had local recurrence with a median follow-up of 25 months [9]. Balloon occlusion of the renal artery might have a place as a standard practice of endophytic tumors in the future. However, it is important to note that this study includes a small cohort and has relatively short follow-up.

Lastly, in a study by De Marini et al., outcomes after MRI-guided PCA of RCC (*n* = 31) reported a primary local recurrence rate of 36% after five years of follow-up. In contrast, De Marini et al. showed a high rate of progression to metastatic disease 16% (*n* = 5), which is difficult to explain. However, the mean tumor size was larger in the cohort studied by De Marini et al. [10] compared to Murray et al. and this study, which could be one explanation for the higher rate of metastatic progression. Therefore, whether MRI guidance is beneficial for PCA is yet to be determined. In the study by Bhagavatula et al. [17], patients with RCC underwent PCA with either CT (*n* = 155) or MRI (*n* = 152) guidance. The study found no differences in key oncological outcomes. MRI-guided PCA of RCC should have a larger role for the treatment of endophytic RCC, given MRI’s excellent soft tissue contrast. However, MRI capacity is limited and this must be weighed against the practicality and lower cost of CT guidance.

From a practical standpoint, it is important to consider the secondary efficacy rate of PCA when evaluating its oncological outcomes. According to Okhunov et al. [18], repeated PCA is a feasible, safe, and less challenging option compared to primary PCA. In this study, repeated PCA was performed in five out of the eight cases of local tumor progression, due to either incomplete ablation or recurrence. Among these, four patients received an additional treatment, which raised the PCA efficacy rate from 86 to 93% and reduced the cumulative incidence estimates of local tumor progression (Fig. 2).

**Fig. 3:**
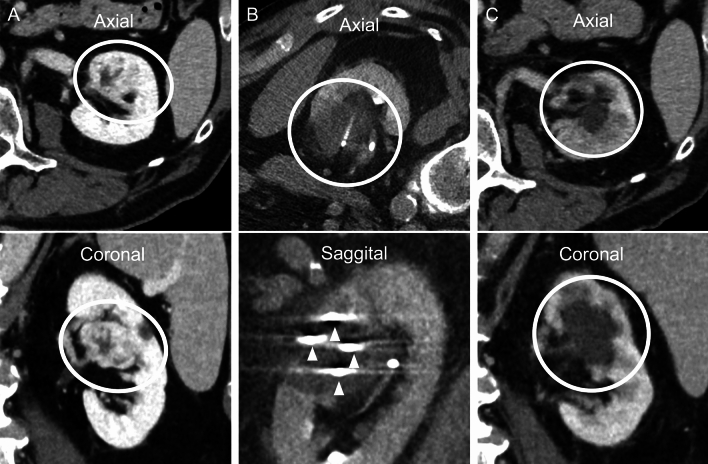
CT images from a 62-year-old male patient with a 38-mm endophytic biopsy-proven clear cell RCC in the left kidney where PCA was performed. **A** Preprocedural CT images with intravenous contrast, venous phase. The tumor is marked with a white circle. **B** Intraprocedural CT images with the patient in an oblique position. The image demonstrates the iceball zone. **C** Venous contrast CT imaging one year after primary PCA shows no residual tumor mass. The ablation zone is marked with a white circle. ^a^Computed tomography (CT). ^b^Renal cell carcinoma (RCC).^c^Percutaneous cryoablation (PCA)

Late local recurrencies after three years were found in both this study and the study by Murray et al. [8], highlighting the importance of long-term follow-up for assessing the true oncological efficacy of PCA in treating endophytic tumors. The results presented exhibit relatively large statistical uncertainties due to the small study size, especially in long-term oncological outcomes. However, this study builds on other data in the literature in supporting the use of PCA for endophytic tumors due to relatively high rates of local control (Fig. 3).

The treatment of endophytic and central RCC has been associated with an increased risk of complications [12, 19].

In our study, we observed a low major complication rate of 5.4% (*n* = 3) according to the CDC system. Murray et al. [8] reported a 10% major complication rate in their cohort. De Marini et al. reported no major complications; however, 23% (*n* = 7) of patients experienced minor (grade ≤ 2) complications, graded by the CDC system.

In a recently published non-randomized prospective study that compared 190 patients with T1a RCC undergoing PCA or PN, both treatments showed similar overall complication rates of 23%, with major complication rates of 3% after PN and 10% after PCA. Notably, in that study, the PCA group treated 16 endophytic tumors, while only one endophytic tumor was treated with PN [4]. This discrepancy might indicate the challenges in treating endophytic RCC.

The complication rates in the literature are heterogeneous. This could be due to a substantial interobserver variability, different treatment modalities, and tumor types [20]. The CDC has been validated for use in the field of urology, but complication classifications related to procedures within interventional radiology, such as the CIRSE system, may better describe the complications after PCA [15, 21].

Ureteral protection during PCA is essential, and this can be done with hydro- or gas dissection, placement of double J-stent, or retrograde pyeloperfusion. At our institution, we do not use pyeloperfusion during PCA. Interestingly, an in vivo experimental study in pigs by Ahmad et al. showed that deep endophytic cryoablation did not affect the renal urothelium [22]. Marion et al. addressed the potential of retrograde pyeloperfusion during PCA. In their RCC cohort, 67 patients with increased risk of ureteral damage underwent pyeloperfusion during PCA and found an acceptable major complication rate given their cohort [23, 24]. With only few complications related to damage of the ureter, it seems that acceptable results can be achieved with the use of hydrodissection and double J-stents as is common practice in our institution.

The present study reflects the treatment of a subset of tumors that are sparsely described in the literature. The data are from a single center and reflect PCA procedural advancements in the period of inclusion. The relatively few tumors in this study do not allow for stratification of variables of interest. Due to the EAU guidelines for the treatment of RCC [3], patients chosen for PCA are often not eligible for extirpative surgery due to comorbidities and/or reduced renal function. Therefore, this study population is not necessarily representative of the average patient with RCC, making mortality and complication rates hard to compare. Furthermore, complications were not divided into early and late complications which could have given better insight in nature of complications after PCA.

## Conclusion

This study demonstrates that PCA of endophytic biopsy-proven T1 RCC is safe with few major complications and excellent local tumor control rates at almost three-year mean follow-up.

### Supplementary Information

Below is the link to the electronic supplementary material.

Supplementary file1 (PDF 9 KB)
