# Supplementary material for: Percutaneous Image-Guided Cryoablation of Endophytic Renal Cell Carcinoma
Source: Cardiovasc Intervent Radiol. 2024 Mar 14;47(4):453–61. doi: 10.1007/s00270-023-03633-5 (PMC10997531; doi:10.1007/s00270-023-03633-5)
Supplement: Supplementary file 1 — Supplementary file1 (PDF 9 KB) [file 270_2023_3633_MOESM1_ESM.pdf]

Electronic Supplementary Material (ESM)

PCA procedure

All PCA procedures were performed in a CT scanner (Siemens Somatom Flash system with 2×128-channel/slice; Siemens Healthcare, Erlangen, Germany). General anesthesia was standard procedure during PCA until September 1, 2017, hereafter most PCA procedures were performed under sedation with intravenous Dexmedetomidine and Remifentanil and injection of local anesthesia (Lidocaine + Bupivacaine).

The type and number of cryo probes were determined before the procedure to ensure that the size of the ice ball would exceed the tumor tissue by a minimum of 5 mm. Anatomical structures adjacent to the treated area were protected by displacement through hydrodisplacement performed with an 18 cm 18 G percutaneous entry thin-wall needle (Cook, Bloomington, IN, USA) with 2% iodine-based saline solution. The cryoprobes were inserted percutaneously under CT-fluoroscopy guidance. An argon-based cryo system (Visual Ice, Boston Scientific, MN, USA) with 17-gauge or 14-gauge sealed cryoprobes being used. During the procedure, sequential CT scans were performed after four and eight minutes in each freeze cycle to evaluate ice ball formation and needle position and to monitor possible complications. PCA was performed with a biphasic freeze-thaw cycle. A standard cycle consisted of 10 minutes freeze; 6.5 minutes passive thaw; 1.5 minutes active thaw; 10 minutes freeze. If the sequential scans showed signs of tumor mass not encompassed by the ice ball, or the ice ball approximating critical tissue, freeze and thaw cycle times were altered accordingly.
